# Heart Rate Fragmentation as a Novel Biomarker of Adverse Cardiovascular Events: The Multi-Ethnic Study of Atherosclerosis

**DOI:** 10.3389/fphys.2018.01117

**Published:** 2018-09-03

**Authors:** Madalena D. Costa, Susan Redline, Roger B. Davis, Susan R. Heckbert, Elsayed Z. Soliman, Ary L. Goldberger

**Affiliations:** ^1^Margret and H. A. Rey Institute for Nonlinear Dynamics in Medicine, Department of Medicine, Beth Israel Deaconess Medical Center, Harvard Medical School, Boston, MA, United States; ^2^Division of Sleep and Circadian Disorders, Departments of Medicine and Neurology, Brigham and Women's Hospital, Boston, MA, United States; ^3^Department of Medicine, Beth Israel Deaconess Medical Center, Harvard Medical School, Boston, MA, United States; ^4^Division of General Medicine and Primary Care, Department of Medicine, Beth Israel Deaconess Medical Center, Harvard Medical School, Boston, MA, United States; ^5^Department of Epidemiology, University of Washington, Seattle, WA, United States; ^6^Department of Epidemiology and Prevention, Epidemiological Cardiology Research Center, Winston-Salem, NC, United States; ^7^Section on Cardiology, Department of Internal Medicine, Wake Forest School of Medicine, Winston-Salem, NC, United States

**Keywords:** aging, alternans, heart failure, heart rate fragmentation, heart rate variability, sino-atrial node, symbolic dynamics, vagal tone

## Abstract

**Background:** A major objective of precision medicine is the elucidation of non-invasive biomarkers of cardiovascular (CV) risk. Recently, we introduced a new dynamical marker of sino-atrial instability, termed heart rate fragmentation (HRF), which outperformed traditional and nonlinear heart rate variability metrics in separating ostensibly healthy subjects from patients with coronary artery disease. Accordingly, we hypothesized that HRF may be a dynamical biomarker of adverse cardiovascular events (CVEs).

**Methods:** This study employed data from a cohort of participants in the Multi-Ethnic Study of Atherosclerosis (MESA), a prospective study of sub-clinical heart disease. Interbeat interval time series (*n* = 1963), derived from the electrocardiographic channel of the polysomnogram study, were analyzed using the newly introduced metrics of fragmentation, as well as traditional heart rate variability (HRV) indices and the short-term detrended fluctuation analysis exponent. Cox regression analysis was used to assess the association between HR dynamic indices and CV outcomes in unadjusted and adjusted models.

**Results:** The mean (± SD) follow-up time was 2.97 ± 0.63 years. In adjusted models, higher fragmentation was significantly associated with incident CVEs (number of events; hazard ratio [95% confidence interval]: *n* = 72, 1.43 [1.16–1.76]) and CV death (*n* = 21; 1.65 [1.15–2.36]). The traditional HRV and the fractal indices were not associated with CVEs or CV death. The most discriminatory fragmentation indices added significant value to Framingham and MESA CV risk indices in all analyses.

**Conclusion:** Our findings show that HRF has promise as a non-invasive, automatable biomarker of CV risk. The basic mechanisms underlying fragmentation remain to be delineated. Its association with incident outcomes raises the possibility of connections to degenerative changes in the multisystem network controlling SAN function.

## 1. Introduction

**Figure 1:**
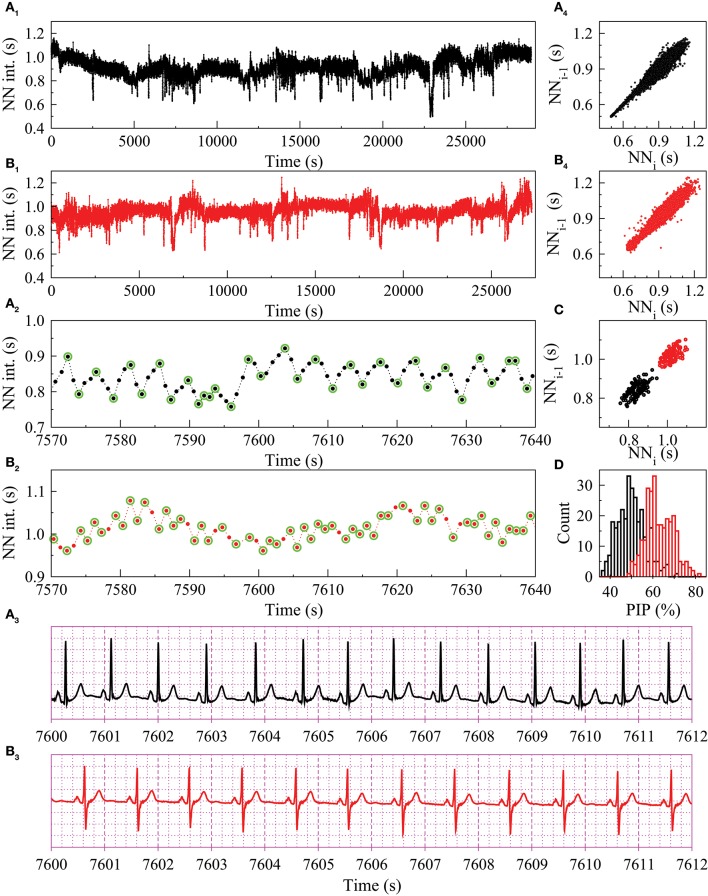
Heart rate dynamics from two MESA participants, **A** with respiratory sinus arrhythmia (black tracings) and **B** with fragmented sinus rhythm (red tracings). Normal-to-normal (NN) sinus interval time series for the entire sleep period **(A**_**1**_**,B**_**1**_**)** and for a 70-second window **(A**_**2**_**,B**_**2**_**)**. Twelve-second ECG recordings **(A**_**3**_**,B**_**3**_**)**. Poincare plots for the entire sleep period **(A**_**4**_**,B**_**4**_**)** and the 70-second NN time series shown in **A**_**2**_ and **B**_**2**_ **(C)**. The green circles highlight the “inflection points,” where the changes in heart rate acceleration sign occur. Histogram of the percentage of inflection points (PIP) calculated with a moving window of 1000 NN intervals **(D)**. Neither participant had prevalent or incident CVEs. However, they were in two different risk categories: the Framingham CVD risk index was 2.4 (1st percentile of MESA participants) for **A** and 15.6 (55th percentile) for **B**. The time series from participant **B** was 30% more fragmented (average PIP = 65%) than the one from participant **A** (average PIP = 50%). Traditional HRV and DFA α_1_ indices were comparable: mean NN interval, 906 and 957 ms; rMSSD, 25.3 and 27.6 ms; pNN50, 5.0 and 6.5%; HF power, 348 and 310 ms^2^; DFA α_1_, 1.05 and 1.18, for participants **A** and **B**, respectively.

This study describes a novel noninvasive biomarker of cardiovascular (CV) risk based on heart rate dynamics. In healthy adults at rest and during sleep, the highest frequency at which the sino-atrial node (SAN) rate fluctuates varies between ~0.15 and 0.40 Hz (Figures 1A_1_–A_4_). These oscillations, referred to as respiratory sinus arrhythmia, are due to vagally-mediated coupling between the SAN and breathing. However, not all fluctuations in heart rate (HR) at or above the respiratory frequency are attributable to vagal tone modulation. Under pathologic conditions, an increased density of reversals in HR acceleration sign, not consistent with short-term parasympathetic control, can be observed (Figures 1B_1_–B_4_). This dynamical biomarker of electrophysiologic instability has recently been identified and termed *heart rate fragmentation* (HRF) (Costa et al., 2017a). A set of metrics (computational probes) for its quantification was also introduced (Costa et al., 2017a,b).

Perhaps the most explicit example of HRF is the subtle supraventricular arrhythmia termed sinus node alternans (Binkley et al., 1995), in which the time between consecutive sinus beats oscillates between two values, short (S) and long (L) following an SLSL pattern. However, HRF includes not only pure (2:1) sinus node alternans but also quasi-periodic and more irregular variants of normal-to normal (NN) alternation. As Figures 1A_3_,B_3_ illustrates, clinical recognition of such patterns is difficult from standard electrocardiograms (ECGs). Traditional heart rate variability (HRV) indices and other metrics such as those derived from Poincaré plots (Figures 1A_4_,B_4_,C) may be also of limited use in the identification of HRF patterns. The basic mechanisms of fragmentation, involving either anomalous sinus beats (Lewis, 1920) or supraventricular ones originating near the SAN, are unresolved (Costa et al., 2017a,b).

The potential importance of HRF is several-fold. First, it produces a high degree of short-term variability that may be mistaken as a marker of healthy vagal control when standard measures of short-term HRV are used. Second, its presence supports the delineation of a new class of biomarkers of cardiac risk. The latter is premised on the conjectured link between HRF and the breakdown in one or more components of the control system (and/or in their interactions) regulating SAN function. Notably, earlier reports of what were first termed “sinus extrasystoles” as well as sinus alternans (Lewis, 1920; Binkley et al., 1995) were from patients who were older or had organic heart disease. Third, the investigation of fragmentation may yield new insights into SAN functionality in health, aging and disease.

In recent studies (Costa et al., 2017a,b) we analyzed annotated Holter recordings [University of Rochester Telemetric Holter ECG Warehouse (THEW)] from healthy subjects and patients with advanced coronary artery disease (CAD) using the newly devised HRF metrics. Fragmentation was found to significantly increase as a function of the participants' age in both the healthy population and the one with CAD. In contrast, most short-term HRV indices did not significantly change with the participants' age in the CAD group. Furthermore, fragmentation was higher in patients with CAD than in healthy subjects, during both estimated awake and sleep periods, while traditional HRV metrics did not discriminate the two groups.

The general motivation for the present study was to assess the potential utility of the novel indices of HRF as predictors of adverse cardiovascular events (CVEs) and CV mortality, using the large Multi-Ethnic Study of Atherosclerosis (MESA). This ongoing prospective cohort study (Bild et al., 2002) was designed to investigate the prevalence, correlates and progression of subclinical cardiovascular disease (CVD) in a multi-ethnic population free of overt clinical CVD at study entry. We specifically hypothesized that HRF would: (1) be positively associated with cross-sectional age; (2) be positively associated with incident CVEs and CV death; and (3) outperform traditional HR dynamical measures. In addition, we also sought to determine if fragmentation metrics added value to prediction tools computed from clinical measures, namely the Framingham Heart Study (D'Agostino et al., 2008) and MESA CV risk indices (McClelland et al., 2015).

## 2. Methods

### 2.1. Study population and data collection

The MESA study has been described in detail previously (Bild et al., 2002). Briefly, over a period of approximately 2-years, starting in July 2000, 6,814 persons between the ages of 45 and 84 years of age without evident clinical CVD were recruited at six field centers in the US. Institutional review boards from each study site approved the conduct of this study, and written informed consent was obtained from all participants.

A sleep ancillary study was conducted in conjunction with MESA's fifth examination (2010–2013). The study enrolled 2,060 participants who underwent unattended, in-home polysomnography (PSG) following a standardized protocol (Redline et al., 1998). The data obtained using the 15-channel Compumedics Somte System (Compumedics LTd., Abbottsville, Australia) were scored at the Brigham and Women's Hospital centralized reading center by trained technicians using published guidelines (Redline et al., 2007). The apnea-hypopnea index (AHI) was calculated based on the average number per hour of sleep of all apneas plus hypopneas associated with ≥3% oxygen desaturation or arousal.

The ECG channels, sampled at 256 Hz, were processed using Compumedics Somte software for detection and classification of the QRS complexes (R-points) as normal sinus, supraventricular premature or ventricular premature complexes. The automated annotations were reviewed by a trained technician, who made appropriate corrections. Both the NN and the R-to-R (RR) interval time series were analyzed in the present study.

Participants with one or more of the following were excluded: poor signal quality (*n* = 35), pacemaker (*n* = 13), atrial fibrillation (AF) at the time of the PSG (*n* = 22), <2 h of combined sleep periods scored as rapid eye movement (REM), stage 1, 2, 3, or 4 (*n* = 16), and <75% normal sinus beats between sleep onset and termination (*n* = 11). Participants with CVEs before the PSG (*n* = 185) and seven others for whom the last recorded follow-up was prior to the PSG were excluded from the analyses of the associations between HR dynamical metrics and incident CVEs. These participants were included in analyses of CV mortality.

### 2.2. Clinical follow-up and event classification

In addition to clinical exams, participants are followed every 9–12 months to inquire about hospital admissions, CV outpatient diagnoses and procedures, and deaths. Discharge diagnosis codes are obtained for all hospitalizations and medical records are obtained when heart failure, myocardial infarction, stroke, or death are reported. For those over age 65 and enrolled in fee-for-service Medicare, claims data are also used to identify diagnosis and procedure codes. Trained personnel abstract any hospital records suggesting possible CVEs, which are then adjudicated by physicians at the coordinating center. Nonfatal endpoints in MESA include congestive heart failure, angina, myocardial infarction, percutaneous coronary intervention, coronary bypass grafting or other revascularization procedure, resuscitated cardiac arrest, peripheral arterial disease, stroke (non-hemorrhagic) and transient ischemia attack (TIA). Cardiovascular deaths, as adjudicated by committee review, included fatalities directly related to stroke or coronary heart disease. For other deaths, the underlying cause are obtained through state or vital statistics departments. The definition and adjudication of these events have been described in detail previously (Bild et al., 2002; Bluemke et al., 2008; Yeboah et al., 2012). The cut-off date for the surveillance period was December 31, 2014.

### 2.3. Fragmentation analysis

Fragmentation analysis was performed for 1963 subjects using both NN and RR interval time series. Fragmentation analysis is described in detail in Costa et al. (2017a,b). Briefly, original interbeat interval time series, {*s*_*i*_}, 1 ≤ *i* ≤ *L* (*L*, time series length) were mapped to a ternary symbolic sequence as follows: “−1” if ΔNN_*i*_ < −4 ms, “0” if −4 ≤ ΔNN_*i*_ ≤ 4 ms, and “1” if ΔNN_*i*_ > 4 ms (Costa et al., 2017b). Note that, since the ECG signals were sampled at 256 Hz, the resolution of the interbeat interval time series is 1/256 ~ 4 ms. Therefore, only NN (or RR) intervals whose difference was >4 ms or <−4 ms were considered different from each other.

Transitions from HR acceleration to HR deceleration (“−1” to “1”) or *vice-versa* (“1” to “−1”), and from HR acceleration or HR deceleration to no change in HR (“−1” to “0,” “1” to “0,”) or *vice-versa* (“0” to “−1,” “0” to “1”) were termed “inflection points.” The percentage of inflection points (PIP) (Costa et al., 2017a) constitutes a measure of HRF reflecting its overall degree of prevalence.

To assess the prevalence of dynamical patterns with increasing degrees of fragmentation, the percentages of sequences of 4 consecutive symbols, *w*_*i*_ = (*s*_*i*_, *s*_*i*+1_, …, *s*_*i*+*l*−1_), 1 ≤ *i* ≤ *L*−*l*+1, termed “words,” with 0, 1, 2, and 3 inflection points were calculated. We refer to these word classes as W_0_, W_1_, W_2_, and W_3_, respectively. The full lexicon that comprises 81 different words is given in Costa et al. (2017b). Words derived from the NN (RR) interval time series were termed NN (RR) words. The words in groups W_0 and W_1 are the least fragmented (most “fluent”), those in groups W_2 and W_3 are the most fragmented.

All fragmentation metrics were calculated for the entire sleep period. In the case of PIP, standard sleep stage periods (awake, rapid eye movement [REM], stages N1, N2, and N3), in addition to periods of awake before sleep onset or after sleep termination were also analyzed. Individuals were included if cumulatively they had at least 2000 NN intervals during a given sleep stage. Thus, the number of participants in each of these sub-analyses was a fraction of the number of participants in the analyses of the entire sleep period. Specifically, the number of participants (the number of those with incident CVEs) for the different periods was: awake, 1379 (61); REM, 1451 (54); N1, 1056 (51); N2, 1695 (70); N3, 791 (23); and awake before sleep onset or after sleep termination, 1449 (58).

### 2.4. Traditional HRV analysis

The following traditional time domain HRV indices (HRV, 1996) were calculated for 1963 subjects using NN interval time series: (1) the average of all NN intervals (AVNN), (2) mean of the standard deviations (SDs) of NN intervals in all 5-min segments (SDNNIDX), (3) the square root of the mean of the squares of differences between adjacent NN intervals (rMSSD), and (4) the percentage of differences between adjacent NN intervals that are greater than 50 ms (pNN50). The following traditional frequency domain HRV indices were calculated: (1) the total spectral power of all NN intervals between 0.15 and 0.4 Hz (HF) and (2) the ratio of low to high frequency power (LF/HF). Each of these metrics was calculated using a 5-min sliding window (without overlap), with more than 150 beats and more than 75% NN intervals, between sleep onset and sleep termination. Power spectrum estimates were obtained using the Lomb periodogram method, which does not require missing data points (due to removal of ectopic, misdetections, and artifact) in a time series to be interpolated (Moody, 1992, 1993). A total of 170,527 windows were analyzed. For each subject, the values from the different windows were averaged. The source code used for these computations is published on The Research Resource for Complex Signals, (PhysioNet) website (Goldberger et al., 2000; Mietus and Goldberger, 2008).

### 2.5. Detrended fluctuation analysis: short-term scaling index

Detrended fluctuation analysis (Peng et al., 1995) was developed to quantify the correlation properties of a time series. The methods is based on the assessment of the slope of the regression line of the logarithm of *F*(*n*) vs. the logarithm of *n*, where *F*(*n*) is the root-mean-square fluctuation of the integrated and detrended data, computed using windows of length *n*. For the analysis of heart rate time series, two indices, α_1_ and α_2_, quantifying short and long-term behavior, respectively, have been proposed. We focused on α_1_, defined as the slope of *F(n) vs. n*, for 4 ≤ *n* ≤ 11 (Pikkujamsa et al., 1999). Time series for which α~0.5 are uncorrelated (random). Those with α > 0.5 and those with α < 0.5 are long-range correlated and anti-correlated, respectively.

Discontinuities in the NN interval time series due to premature beats, misdetections and artifact were dealt with in the following way. If the gap was < 3 s (typically due to the removal of an ectopic beat), an interpolated beat was inserted. If the gap was wider (typically due to ECG artifact that prevents accurate detection of the peak of the QRS complexes), then the segments that preceded and followed the gap, were “stitched” together. The α_1_ exponent was calculated for non-overlapping segments of 1,000 intervals and the results were averaged. The source code used in these computations is published on the PhysioNet website (Goldberger et al., 2000; Mietus et al., 2001).

### 2.6. Statistical analysis

Continuous variables are summarized as median, first and third quartiles, unless otherwise indicated. Categorical variables are presented as numbers and percentages.

The associations between independent variables and both incident CVEs and CV mortality were assessed using Cox proportional hazard analysis. Efron's method (Efron, 1977) was used to handle ties. Failure time in the individuals with incident CVEs was the time between the PSG study and the time of event diagnosis. For participants without CVEs, the failure time was the time between the PSG study and the latest follow-up, death, or loss to follow-up. Statistical significance was set at a *p*-value < 0.05. The independent variables were: the fragmentation indices, PIP, W_0_, W_1_, W_2_, and W_3_, derived from both NN and RR interval time series; the HR dynamical indices: AVNN, SDNNIDX, rMSSD, pNN50, HF, and HF/LF; and the short-term fractal index DFA α_1_.

Both unadjusted (Model 1) and adjusted models were considered. Adjustments included: (i) traditional CV risk factors: age, sex, systolic blood pressure, total cholesterol, high density lipoprotein (HDL) cholesterol, current smoking status, hypertension medication, diabetes and lipid lowering medication (Model 2); (ii) the Framingham Heart Study 10-year risk index (D'Agostino et al., 2008) (Model 3), and (iii) the MESA CV risk index without coronary calcification (Model 4).

Standardized hazard ratios (per one-SD increase in the independent variable) were calculated with associated 95% confidence intervals (CI). The assumption of proportional hazards was tested using a global test based on Schoenfeld residuals (Grambsch and Therneau, 1994). No violations were noted. The predictive power of the survival models was assessed using Harrell's C statistic. The likelihood ratio test was used to compare the fit of two nested models (null model vs. null model + HR dynamical metric). The three null models were models 2, 3, and 4 described above. The null hypothesis for each of the likelihood ratio tests was that the two nested models fitted the data equally well. Rejection of the null hypothesis implied that the larger model fitted the data better, indicating that a given HR dynamical metric added value to the null model.

Linear regression models with a quadratic term were used to describe the possible nonlinear (U-shaped) relationship between: i) HRF and short-term HRV indices (example of a model with PIP and rMSSD, $\text{PIP}=\beta_{1}*\text{ln}\left(\text{rMSSD}\right)+\beta_{2}*{\left[\text{ln}\left(\text{rMSSD}\right)\right]}^{2}+\alpha$, where α is a constant); and ii) short-term HRV indices and the participants' age.

In all analyses, the variables W_0_, rMSSD, pNN50, SDNN, or HF were logarithmically transformed since the models with these log-transformed variables fitted the data better than those with untransformed ones. In all other cases, the analyses were performed using untransformed variables.

## 3. Results

Over a median follow-up period of 1,080 [916–1,259] (median [Q_1_–Q_3_]) days after the PSG study, 72 out of the 1,771 participants without prevalent CVEs suffered their first adverse event: myocardial infarction (*n* = 16), resuscitated cardiac arrest (*n* = 1), angina (*n* = 14), percutaneous coronary intervention (*n* = 21), coronary bypass graft (*n* = 3), other revascularization (*n* = 6), congestive heart failure (*n* = 10), peripheral vascular disease (*n* = 8), transient ischemic attack (*n* = 5), CV death (*n* = 14) or stroke (non-hemorrhagic brain infarction, *n* = 17). From a total of 1,963 participants (1,771 without and 192 with prevalent CVEs), 21 died of CVD.

Characteristics of MESA participants without and with a CVE during follow-up are summarized in Table 1. Individuals who developed CVEs were older and more likely to be male and have diabetes. They tended to have higher seated HR and higher systolic blood pressure. In addition, this risk group tended to have lower sleep efficiency and a higher apnea-hypopnea index. The differences between those who died and did not were qualitatively similar to the differences between those with and without incident CVEs.

**Table 1:** Characteristics of MESA participants without and with a CVE during follow-up.

**Variable**	**No incident CVEs**	**Incident CVEs**	***p*-value**
	**(*N* = 1699)**	**(*N* = 72)**	
Age (years)	66.0 [60.0–74.0]	72.5 [62.5–78.0]	**<0.001**
Male, *n*(%)	755 (44)	46 (64)	**0.001**
Race			0.577
Caucasian, *n*(%)	618 (36)	23 (32)	
Chinese, *n*(%)	218 (13)	7 (10)	
African American, *n*(%)	467 (27)	21 (29)	
Hispanic, *n*(%)	396 (23)	21 (29)	
BMI (kg/m^2^)	27.8 [24.4–31.7]	27.1 [25.0–30.7]	0.646
Waist circumference (cm)	97.8 [88.8–107.4]	99.0 [93.5–106.1]	0.280
Seated Heart rate (bpm)	63.5 [57.0–70.0]	64.5 [59.3–74.3]	0.081
Systolic blood pressure (mmHg)	119 [109–134]	123 [109–140]	0.073
Diastolic blood pressure (mmHg)	68.0 [62.0–74.5]	68.8 [61.8–75.5]	0.111
Total cholesterol / HDL	3.38 [2.78–4.17]	3.35 [2.56–4.36]	0.355
Triglycerides (mg/dL)	96 [70–131]	97 [69–130]	0.473
Current smoker, *n*(%)	104 (6)	7 (10)	0.230
Antihypertensive medication, *n*(%)	829 (49)	42 (58)	0.113
Diabetes, *n*(%)	290 (17)	20 (28)	**0.022**
Lipid lowering medication, *n*(%)	568 (33)	28 (39)	0.337
Oral hypoglycemic agents, *n*(%)	219 (13)	17 (24)	**0.009**
Total sleep time (min)	370 [316–418]	352 [293–408]	0.066
Sleep efficiency (%)	78.8 [69.6–85.9]	72.8 [64.8–82.1]	**0.003**
Apnea-hypopnea index	17.4 [8.83–31.9]	26.2 [13.4–40.6]	**0.012**
Central apnea index	0 [0–0.27]	0 [0–0.38]	0.969

*Values presented are the population median, first and third quartiles for continuous variables and the number of participants and its percentage for categorical variables. The Student t-test and the χ^2^ test were used for assessing between-group (incident CVEs vs. no incident CVEs) differences in continuous and categorical variables, respectively. Abbreviations: BMI, body mass index; CVE, cardiovascular event; HDL, high density lipoprotein cholesterol; SD, standard deviation. Boldface is used to highlight p-values < 0.05, i.e., statistically significant differences between groups with and without CVEs*.

### 3.1. Relationship of HRF, traditional HRV and nonlinear indices with the participants' age

**Figure 2:**
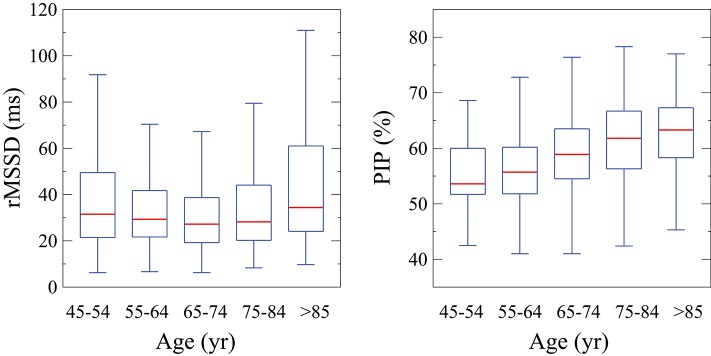
Tukey boxplots of ln(rMSSD) and PIP for participants in successive age groups. PIP, percentage of inflection points; rMSSD, root mean square of the successive differences.

All fragmentation indices, derived from either NN or RR interval time series, were significantly associated with the participants' age. The Pearson correlation coefficients (ρ) for PIP, W_0_, W_1_, W_2_, and W_3_ were 0.35, −0.17, −0.31, 0.10, and 0.35, respectively. PIP (Figure 2) and the percentages of fragmented words W_2_ and W_3_ increased with the participants' age at the estimated rates of 0.28, 0.09, and 0.35% per year of age, respectively. The percentages of fluent words W_0_ and W_1_ decreased with the participants' age at the rates of −0.06 and −0.39% per year, respectively. Slightly higher rates of change were observed for the indices derived from RR interval time series (not shown).

The short-term traditional HRV indices, rMSSD, pNN50 and HF, did not vary linearly with the participants' age. Instead, the association between age and short-term variability depended on the participants' age itself. Figure 2 illustrates, using the representative example of rMSSD, how the amount of short-term variability varied across different age groups. Variability was higher in both the lowest (<55 years) and highest (≥85 years) age groups compared to intermediate ones (U-shape relationship).

The short-term fractal index, α_1_ showed a small but significant decrease with cross-sectional age. The Spearman correlation coefficient was −0.18 (*p* = 0.000).

### 3.2. Unadjusted analyses of risk of incident CVEs

**Figure 3:**
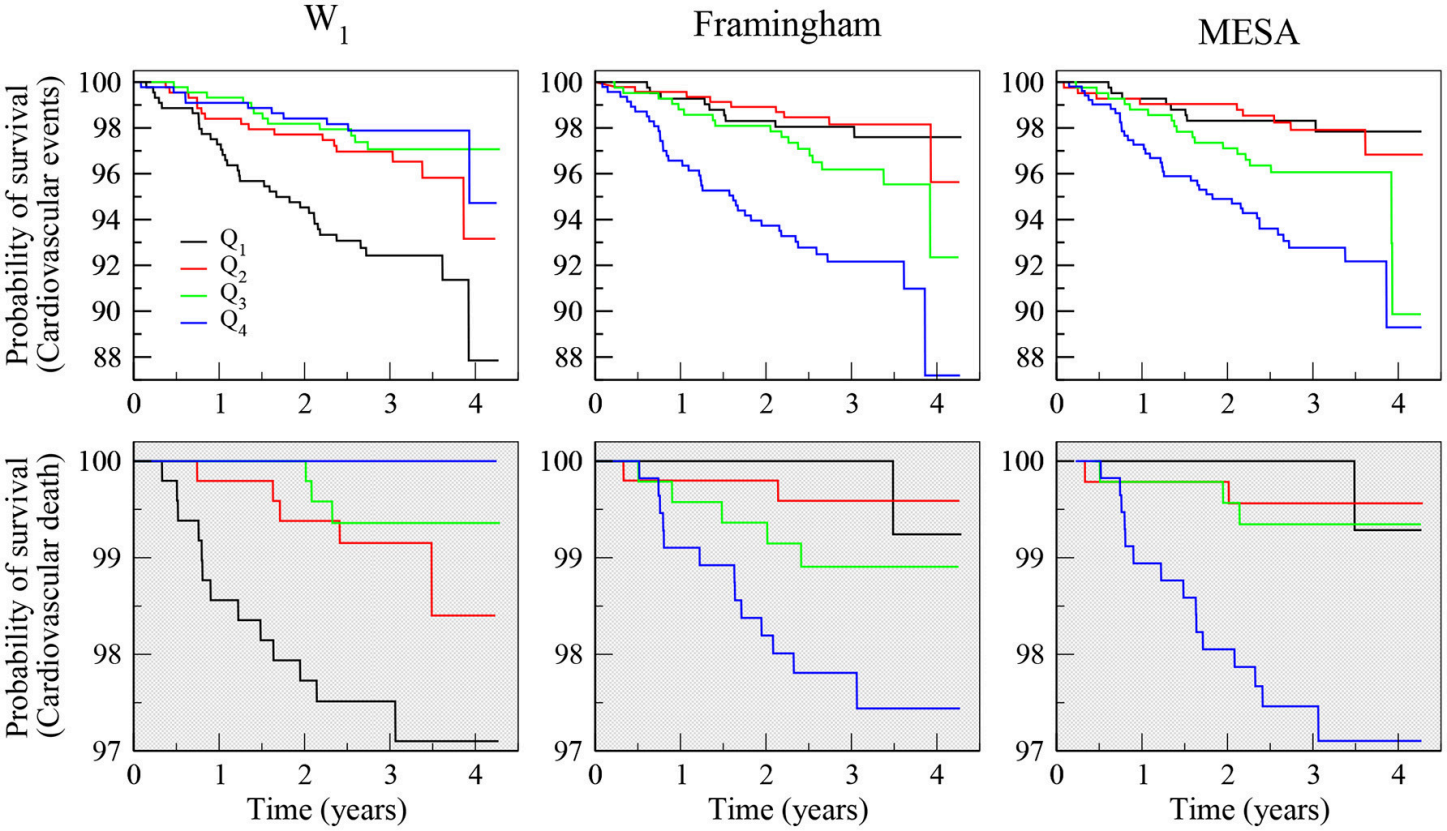
Kaplan-Meier survival curves of analyses of incident CVEs (top panels) and CV mortality (bottom panels), showing the percentage of symbolic words with one inflection point (W_1_) derived from RR interval time series (left panels), the Framingham (middle panels) and MESA (right panels) CV risk indices. Q_1_–Q_4_ indicate first to fourth quartiles. Note that participants in the highest quartile of the Framingham and MESA risk indices had the worst prognosis and those in the highest quartile of word class W_1_ (more fluent, less fragmented) had the best prognosis.

All fragmentation indices, calculated from both the NN and the RR time series, were significantly associated with incident events (Table 2: Model 1). The association was positive for fragmented words, W_2_ and W_3_, as well as PIP, and negative for fluent (less fragmented) words, W_0_ and W_1_. The most discriminatory of the fragmentation indices was the word W_1_ derived from the RR interval time series. A one-SD increase in W_1_ was associated with a 44% (95% CI: 28–66%) decrease in the rate of CVEs. This variable performed comparably to the Framingham Heart Study and MESA CV risk indices (Figure 3, top panels).

**Table 2:** Association of fragmentation, traditional HRV and short-term fractal indices with incident CVEs in unadjusted and adjusted models for standard risk factors.

			**Model 1 (Unadjusted)**		**Model 2 (Traditional factors)**		
	**Variable**	**Median (Q_1_–Q_3_)**	**HR_s_ (95% CI)**	**C-index**	**HR_s (95% CI)**	**C-index**	**LR-test**
Fragmentation using NN	PIP (%)	58.0 (53.4–62.9)	1.60 (1.31–1.96)	0.648	1.43 (1.15–1.78)	0.712	**0.002**
	*W*_0_ (%)	4.50 (2.72–7.02)	0.76 (0.61–0.94)	0.574	0.75 (0.60–0.94)	0.705	**0.014**
	*W*_1_ (%)	31.7 (24.1–39.3)	0.59 (0.46–0.75)	0.655	0.67 (0.51–0.87)	0.712	**0.002**
	*W*_2_ (%)	44.1 (39.6–50.4)	1.26 (1.01–1.58)	0.574	1.30 (1.02–1.65)	0.697	**0.037**
	*W*_3_ (%)	15.6 (11.2–22.1)	1.39 (1.20–1.62)	0.626	1.25 (1.05–1.49)	0.697	**0.027**
Fragmentation using RR	PIP (%)	58.1 (53.6–63.1)	1.61 (1.32–1.96)	0.651	1.43 (1.15–1.78)	0.712	**0.002**
	*W*_0_ (%)	4.46 (2.67–6.92)	0.73 (0.59–0.90)	0.574	0.72 (0.58–0.90)	0.708	**0.005**
	*W*_1_ (%)	31.3 (23.9–39.1)	0.56 (0.44–0.72)	0.664	0.64 (0.49–0.83)	0.720	**0.001**
	*W*_2_ (%)	44.3 (39.8–50.5)	1.35 (1.08–1.68)	0.580	1.40 (1.10–1.78)	0.706	**0.007**
	*W*_3_ (%)	15.9 (11.5–22.4)	1.37 (1.17–1.60)	0.612	1.21 (1.01–1.46)	0.693	0.062
Traditional HRV and short-term fractal	AVNN (ms)	942 (861–1033)	0.86 (0.68–1.09)	0.529	0.84 (0.65–1.07)	0.688	0.155
	SDNNIDX (ms)	46.9 (35.4–61.7)	0.90 (0.71–1.14)	0.529	0.89 (0.71–1.12)	0.685	0.312
	rMSSD (ms)	28.6 (20.6–42.2)	0.96 (0.75–1.21)	0.539	0.91 (0.73–1.14)	0.683	0.419
	pNN50 (%)	6.03 (1.71–16.5)	0.83 (0.67–1.04)	0.545	0.85 (0.68–1.05)	0.687	0.137
	HF (× 10^3^ms^2^)	3.75 (19.7–7.43)	0.98 (0.77–1.23)	0.527	0.94 (0.75–1.17)	0.684	0.565
	LF/HF (unitless)	2.03 (1.25–3.53)	0.85 (0.68–1.07)	0.541	0.91 (0.71–1.15)	0.693	0.413
	DFA α_1_ (unitless)	1.18 (1.00–1.35)	0.95 (0.75–1.19)	0.511	0.98 (0.77–1.25)	0.689	0.889

*Model 1: unadjusted. Model 2: adjusted for the traditional risk factors: age, sex, systolic blood pressure, total cholesterol, HDL cholesterol, current smoking status, hypertension medication (including beta blockers, calcium-channel blockers, angiotensin antagonists, and angiotensin antagonists plus diuretics), diabetes and lipid lowering medication. Values presented are standardized hazard ratios (HR_s), 95% confidence intervals (95% CI), Harrell's C statistic (C-index) and the p-value for the likelihood ratio test (LR-test) of the null hypothesis that the addition of a dynamical measure (fragmentation, traditional HRV or DFA α_1_ metric) to a model with the traditional risk factors did not improve the fit of the data. The numbers of participants/events in the analyses of the models 1 and 2 were 1771/72 and 1702/71, respectively. Abbreviations: Q_1_, first quartile; Q_3_, third quartile; HRV, heart rate variability; CVE, cardiovascular event; HDL, high density lipoprotein; PIP, percentage of inflection points; W_0, W_1, W_2, W_3, percentage of words with 0, 1, 2, and 3 inflection points, respectively; AVNN, average value of the NN intervals; SDNNIDX, mean of the standard deviations of NN intervals in all 5-min segments; rMSSD, root mean square of the successive differences; pNN50, percentage of differences between successive NN intervals above 50 ms; HF, high frequency spectral power; LF/HF, ratio of low to high frequency power; DFA α_1_, detrended fluctuation analysis short-term fractal exponent. Boldface is used to highlight p-values < 0.05*.

None of the traditional time (AVNN, SDNNIDX, rMSSD, pNN50) and frequency domain (HF, LF/HF) HRV variables was significantly associated with incident events. DFA α_1_ was also not associated with incident events.

### 3.3. Analyses of risk of incident CVEs adjusted for traditional risk factors

The models with each of the fragmentation and traditional HRV variables were adjusted for standard CV risk factors: age, sex, systolic blood pressure, total cholesterol, HDL cholesterol, current smoking status, hypertension medication, diabetes and lipid lowering medication. In these analyses (Table 2: Model 2), all fragmentation indices remained significantly associated with incident CVEs. For example, a one-SD increase in PIP was associated with a 43% (15–78%) increase in the rate of incident CVEs. In addition, all fragmentation indices, with the exception of W_3_ calculated from the RR interval time series, added significant value to the model with only the risk factors. In contrast, none of the traditional time (AVNN, SDNNIDX, rMSSD, pNN50) and frequency domain (HF, LF/HF) HRV variables was significantly associated with incident events. DFA α_1_ was also not associated with incident events.

Age and sex were the only covariates significantly associated with incident CVEs in the adjusted models that included a fragmentation index. Specifically, for a model with PIP, the standardized hazard ratios for each of the covariates were: i) age (SD = 9.1 years), 1.33 (95% CI: 1.03–1.73), *p* = 0.029; ii) sex, 1.95 (1.15–3.30), *p* = 0.013; iii) total cholesterol (SD = 36.6 mg/dl), 0.91 (0.69–0.21), *p* = 0.533; iv) HDL (SD = 16.3 mg/dl), 0.87 (0.65–1.14), *p* = 0.311; v) diabetes, 1.39 (0.78–2.46), *p* = 0.260; vi) smoking status, 1.66 (0.74–3.71), *p* = 0.215; vii) hypertension medication, 1.02 (0.61–1.71), *p* = 0.938; viii) systolic blood pressure (SD = 20.3 mmHg), 1.12 (0.89–1.42), = 0.342; and ix) lipid lowering medication, 0.97 (0.75–1.25), *p* = 0.786.

The results did not qualitatively change after further adjusting the analyses for each of the following variables, singly or in combination: race, body mass index, waist circumference, diastolic blood pressure, seated heart rate, use of hypoglycemic agents, total sleep time, sleep efficiency, the apnea-hypopnea index, measures of coronary artery calcification (McClelland et al., 2015) (total Agatston calcium score) and carotid plaque (Gepner et al., 2015, 2017).

For all sleep stages, including awake during sleep, and awake before sleep onset and/or sleep termination, the degree of HRF, measured by PIP, was positively associated with risk of incident CVEs in fully adjusted models. The levels of significance varied slightly per stage. Specifically, the standardized hazard ratios and 95% CI were: REM [1.34 (1.01–1.77)], stage N1 [1.51 (1.13–2.00)], stage N2 [1.36 (1.08–1.70)], and stage N3 [1.55 (0.99–2.43)], awake [1.68 (1.27–2.22)] and awake before sleep onset and/or sleep termination [1.71 (1.34–2.19)].

### 3.4. Analyses of risk of incident CVEs adjusted for the framingham and MESA CV risk indices

In general, the fragmentation indices remained significantly associated with the risk of CVEs in models adjusted for the Framingham and the MESA CV risk indices (Table 3). Specifically, increased fragmentation, that is, higher PIP, lower percentages of fluent words W_0_ and W_1_, and higher percentages of fragmented words W_2_ and W_3_, were significantly associated with increased risk of events. Neither the traditional HRV measures nor DFA α_1_ showed significant associations with incident CVEs.

**Table 3:** Association of fragmentation, traditional HRV and short-term fractal indices with incident CVEs in models adjusted for the Framingham (model 3) and MESA (model 4) risk indices.

		**Model 3 (Framingham)**			**Model 4 (MESA)**		
	**Variable**	**HR_**s**_ (95% CI)**	**C-index**	**Lr-test**	**HR_**s**_ (95% CI)**	**C-index**	**LR-test**
Fragmentation using NN	PIP	1.43 (1.16–1.76)	0.698	**0.001**	1.44 (1.17–1.78)	0.689	**0.001**
	*W*_0_	0.78 (0.63–0.96)	0.684	**0.022**	0.77 (0.62–0.95)	0.670	**0.017**
	*W*_1_	0.66 (0.52–0.86)	0.699	**0.001**	0.64 (0.50–0.83)	0.695	**0.001**
	*W*_2_	1.24 (0.99–1.56)	0.677	0.070	1.29 (1.02–1.63)	0.678	**0.035**
	*W*_3_	1.27 (1.08–1.50)	0.688	**0.011**	1.27 (1.07–1.49)	0.680	**0.013**
Fragmentation using RR	PIP	1.44 (1.17–1.77)	0.698	**0.001**	1.45 (1.18–1.78)	0.689	**0.001**
	*W*_0_	0.74 (0.60–0.92)	0.688	**0.007**	0.74 (0.60–0.91)	0.670	**0.006**
	*W*_1_	0.64 (0.49–0.82)	0.707	**<0.001**	0.61 (0.47–0.80)	0.703	**<0.001**
	*W*_2_	1.33 (1.06–1.67)	0.687	**0.015**	1.40 (1.11–1.76)	0.681	**0.006**
	*W*_3_	1.24 (1.05–1.48)	0.681	**0.026**	1.24 (1.04–1.47)	0.670	**0.031**
Traditional HRV and short-term fractal	AVNN	0.84 (0.67–1.07)	0.671	0.156	0.86 (0.67–1.09)	0.674	0.195
	SDNNIDX	0.91 (0.72–1.13)	0.668	0.378	0.89 (0.71–1.11)	0.677	0.311
	rMSSD	0.94 (0.75–1.18)	0.667	0.584	0.92 (0.73–1.14)	0.675	0.429
	pNN50	0.86 (0.69–1.06)	0.669	0.167	0.84 (0.68–1.04)	0.672	0.121
	HF	0.96 (0.77–1.20)	0.668	0.750	0.94 (0.75–1.17)	0.674	0.588
	LF/HF	0.87 (0.71–1.11)	0.671	0.296	0.90 (0.72–1.13)	0.681	0.380
	DFA α_1_	0.96 (0.77–1.20)	0.664	0.714	0.99 (0.79–1.23)	0.678	0.899

*Model 3: adjusted for the Framingham CV risk index (D'Agostino et al., 2008). Model 4: adjusted for the MESA CV risk index. Values presented are standardized hazard ratios (HR_s), 95% confidence intervals (95% CI), Harrell's C statistics (C-index) and the p-value for the likelihood ratio test (LR-test) of the null hypothesis that the addition of an HR dynamical metric (fragmentation, traditional HRV or DFA α_1_) to a model with the Framingham or MESA risk indices did not improve the fit of the data. The numbers of participants/events in the analyses of models 3 and 4 were 1767/72 and 1672/71, respectively. Abbreviations: HRV, heart rate variability; CVE, cardiovascular event; CV, cardiovascular; PIP, percentage of inflection points; W_0, W_1, W_2, W_3, percentage of words with 0, 1, 2, and 3 inflection points; AVNN, average value of the normal-to-normal sinus (NN) intervals; SDNNIDX, mean of the standard deviations of NN intervals in all 5-min segments; rMSSD, root mean square of the successive differences; pNN50, percentage of differences between successive NN intervals above 50 ms; HF, high frequency spectral power; LF/HF, ratio of low to high frequency power; DFA α_1_, detrended fluctuation analysis short-term fractal exponent. Boldface is used to highlight p-values < 0.05*.

The risk indices in each of these models were also significantly associated with incident CVEs. Specifically, a one-SD increase in the Framingham and in the MESA risk indices, was associated with 80% (95% CI: 43–125%), and 55% (33–81%) increase in the rate of adverse CVEs, respectively. Harrell's C statistic was 0.666 and 0.678 for the Framingham and MESA risk indices, respectively. Overall the best model, with a Harrell's C statistic of 0.703, was the one that combined the word group W_1_ derived from RR intervals, with the MESA risk index.

Models incorporating the Framingham risk index and any of the fragmentation measures, except W_2_ derived from the NN interval time series, performed significantly better than the Framingham index itself. Similarly, all models that included any of the fragmentation measures in addition to the MESA risk index performed significantly better than the MESA index itself.

The traditional HRV variables and α_1_ were not significantly associated with risk of incident CVEs either in unadjusted or adjusted models. Adding one of these indices to a model with a fragmentation index did not improve its performance.

### 3.5. Analyses of risk of CV death: unadjusted and adjusted for the framingham and MESA risk indices

Higher PIP and lower percentage of W_1_ words were significantly associated with increased risk of CV death in unadjusted analyses as well as in analyses adjusted for the Framingham and the MESA CV risk indices (Table 4, Figure 3). Specifically, a one-SD (~7%) increase in PIP derived from the analysis of NN interval time series was associated with an increase in the rate of CV death of 89% (95% CI: 34–168%) in unadjusted models and of 65% (15–136%) and 67% (19–136%) in models adjusted for the Framingham and the MESA risk indices, respectively. A one-SD (~11%) increase in the percentage of W_1_ (“fluent” or least fragmented) words, also derived from the analysis of NN interval time series, was associated with a decrease in the rate of CV death of 59% (37–75%) in unadjusted models and of 52% (21–71%) and 55% (25–72%) in models adjusted for Framingham and the MESA risk indices, respectively. Similar results were obtained from the analyses of the RR interval time series. Further adjusting the models by prevalent CVD did not change the significance of the associations between the fragmentation metrics and risk of CV death.

**Table 4:** Association of fragmentation, traditional HRV and short-term fractal indices with CV death in models adjusted for the Framingham and MESA CV risk indices.

		**Model 1 (Unadjusted)**		**Model 2 (Framingham)**			**Model 3 (MESA)**		
	**Variable**	**HR_s (95% CI)**	**C-index**	**HR_s_ (95% CI)**	**C-index**	**LR-test**	**HR_s_ (95% CI)**	**C-index**	**LR-test**
Fragmentation using NN	PIP	1.89 (1.34–2.68)	0.726	1.65 (1.15–2.36)	0.805	**0.011**	1.67 (1.19–2.36)	0.829	**0.007**
	*W*_0_	0.73 (0.49–1.07)	0.583	0.77 (0.52–1.13)	0.771	0.185	0.72 (0.49–1.06)	0.814	0.105
	*W*_1_	0.41 (0.25–0.67)	0.747	0.48 (0.29–0.79)	0.819	**0.003**	0.45 (0.28–0.75)	0.829	**0.001**
	*W*_2_	1.47 (0.98–2.19)	0.623	1.44 (0.95–2.19)	0.783	0.089	1.52 (0.99–2.33)	0.816	0.062
	*W*_3_	1.51 (1.18–1.93)	0.704	1.36 (1.04–1.76)	0.782	0.055	1.37 (1.06–1.76)	0.808	**0.044**
Fragmentation using RR	PIP	1.87 (1.33–2.63)	0.735	1.63 (1.14–2.34)	0.805	**0.013**	1.65 (1.17–2.32)	0.832	**0.009**
	*W*_0_	0.71 (0.49–1.03)	0.593	0.75 (0.51–1.09)	0.775	0.145	0.71 (0.49–1.03)	0.816	0.085
	*W*_1_	0.38 (0.23–0.63)	0.762	0.44 (0.26–0.74)	0.827	**0.001**	0.43 (0.26–0.71)	0.838	**0.001**
	*W*_2_	1.65 (1.12–2.43)	0.643	1.66 (1.10–2.50)	0.796	**0.019**	1.74 (1.15–2.65)	0.813	**0.012**
	*W*_3_	1.46 (1.13–1.90)	0.696	1.31 (0.99–1.74)	0.775	0.104	1.32 (1.00–1.74)	0.804	0.086
Traditional HRV and short-term fractal	AVNN	0.72 (0.46–1.15)	0.565	0.71 (0.45–1.11)	0.768	0.120	0.66 (0.42–1.05)	0.788	0.075
	SDNNIDX	0.75 (0.48–1.16)	0.558	0.77 (0.51–1.16)	0.767	0.211	0.75 (0.49–1.15)	0.788	0.184
	rMSSD	0.90 (0.58–1.41)	0.536	0.88 (0.58–1.33)	0.756	0.536	0.88 (0.58–1.34)	0.793	0.551
	pNN50	0.77 (0.51–1.14)	0.564	0.79 (0.54–1.17)	0.762	0.250	0.77 (0.52–1.14)	0.775	0.202
	HF	0.89 (0.57–1.39)	0.522	0.88 (0.59–1.33)	0.755	0.543	0.89 (0.59–1.36)	0.795	0.591
	LF/HF	0.71 (0.47–1.08)	0.552	0.77 (0.51–1.16)	0.754	0.219	0.73 (0.49–1.10)	0.778	0.136
	DFA α_1_	0.79 (0.52–1.20)	0.545	0.84 (0.56–1.16)	0.754	0.386	0.81 (0.54–1.20)	0.802	0.294

*Model 1: unadjusted. Model 2: adjusted for the Framingham CV risk index (D'Agostino et al., 2008). Model 3: adjusted for the MESA CV risk index. The number of participants/events in models 1, 2, and 3 was 1963/21, 1958/21, and 1856/21, respectively. Values presented are standardized hazard ratios (HR_s), 95% confidence intervals (95% CI), Harrell's C statistics (C-index) and the p-value for the likelihood ratio test (LR-test) of the null hypothesis that the addition of a dynamical measure (fragmentation, traditional HRV or DFA α_1_ metric) to a model with one of the risk indices did not improve the fit of the data. Abbreviations: HRV, heart rate variability; CV, cardiovascular; PIP, percentage of inflection points; W_0, W_1, W_2, W_3, percentage of words with 0, 1, 2, and 3 inflection points; AVNN, average value of the normal-to-normal sinus (NN) intervals; SDNNIDX, mean of the standard deviations of NN intervals in all 5-min segments; rMSSD, root mean square of the successive differences; pNN50, percentage of differences between successive NN intervals above 50 ms; HF, high frequency spectral power; LF/HF, ratio of low to high frequency power; DFA α_1_, detrended fluctuation analysis short-term fractal exponent. Boldface is used to highlight p-values < 0.05*.

Lower percentages of fluent words W_0_ were associated with increased risk of CV death in all models. However, these associations were weaker than those with word W_1_. The percentages of word W_2_ and W_3_, the most fragmented, were positively associated with the risk of CV death in all models. However, the significance of the associations depended on the particular model (Table 4). A one-SD increase in the Framingham (~9%) and in the MESA risk (~6%) indices was associated with 137% (95% CI: 48–278%) and 62% (35–96%) increase in the rate of CV death, respectively. Harrell's C statistic for the former and latter models was 0.749 and 0.797, respectively. Both variables, PIP and W_1_, calculated from NN and RR interval time series, added significant information to models with either of these risk indices. The results for word groups W_0_, W_2_, and W_3_ depended on the particular model. Overall, the best model, with a Harrell's C statistic of 0.838, was the one that combined the word W_1_, derived from RR intervals, with the MESA risk index.

The traditional HRV variables and α_1_ were not significantly associated with risk of CV death either in unadjusted or adjusted models. Adding one of these indices to a model with a fragmentation index did not improve its performance.

### 3.6. Relationship of HRF with short-term traditional HRV indices

**Figure 4:**
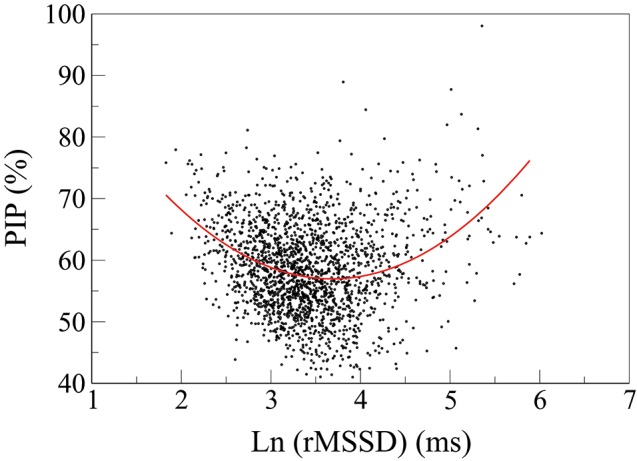
Scatter plot of PIP vs. the natural logarithm of rMSSD. The fitting line is described by the equation: PIP = −26.2^*^ln(rMSSD)+3.54^*^[ln(rMSSD)]^2^+105.3. The 95% CI for the 1st, 2nd and 3rd terms are: (−30.0 − −22.5), (3.03–4.05) and (98.5–112.1), respectively. PIP, percentage of inflection points; rMSSD, root mean square of the successive differences.

Nonlinear (U-shape) relationships were found between fragmentation indices and traditional HRV measures of short-term variability. Figure 4 shows one representative example, the relationship between PIP and ln(rMSSD). For the first three quartiles of rMSSD values, (i.e., for rMSSD values below the 75th percentile of rMSSD, specifically, ln(rMSSD) < 3.7 ms), the degree of fragmentation and the amount of short-term variability were inversely correlated. In the upper quartile of rMSSD values, however, the degree of fragmentation and the amount of short-term variability were positively associated. Qualitatively similar results were found for pNN50 and HF power.

## 4. Discussion

The present investigation was designed to test the association of quantitative measures of HRF, a newly defined property of short-term sino-atrial rhythm dynamics, with adverse CV outcomes in MESA, a large ongoing multicenter study of individuals recruited from the general community. The key findings of this study were that: (1) increased HRF was significantly associated with risk of incident CVEs and CV mortality; (2) measures of fragmentation added value to Framingham and MESA risk prediction indices; and (3) traditional metrics of short-term HRV as well as a nonlinear index (DFA α_1_) were not associated with incident CVEs or CV death.

The development of the concept of fragmentation and its quantitative metrics was motivated in part by an apparent paradox in the results of traditional time and frequency domain analyses of a number of studies (de Bruyne et al., 1999; Stein et al., 2005; Huikuri and Stein, 2012; Drawz et al., 2013; Costa et al., 2017a; Raman et al., 2017). While the mechanisms of the relatively slow (i.e., below the respiratory frequency) variations in HR are attributable to complex interactions between the parasympathetic and the sympathetic branches of the autonomic nervous system, faster variations, in the range of 0.15−0.40Hz, are mainly attributed to vagal tone modulation (HRV, 1996). Therefore, short-term (high frequency) measures of HR dynamics, such as rMSSD, pNN50, and HF power, are typically interpreted as surrogate measures of cardiac vagal tone. In contexts where cardiac vagal tone modulation is known to be diminished, for example, with advanced aging and established CVD, these “vagal” measures are expected to be lower. In fact, a monotonic decrease in high frequency variability with increasing age has been reported in multiple cross-sectional studies of ostensibly healthy adults (Pikkujamsa et al., 1999; Bonnemeier et al., 2003; Costa et al., 2017a). Furthermore, the association between extremely low variability and adverse outcomes is well-documented (HRV, 1996; Huikuri and Stein, 2012; O'Neal et al., 2016).

However, in certain cases (de Bruyne et al., 1999; Stein et al., 2008; Almeida-Santos et al., 2016; Raman et al., 2017; Wdowczyk et al., 2018) a paradoxical increase in short-term HR fluctuations was observed in contexts where reduced vagal tone would have been expected based on age and/or advanced heart disease. In the present study, we observed a U-shaped relationship between traditional short-term HRV measures and cross-sectional age (Figure 2). From approximately ages 45–65 years the amount of short-term variability decreased. Subsequently, variability increased despite the well-known decrease in cardiac vagal tone modulation with advancing age. *These results provide further evidence that in cohorts of middle-aged to elderly individuals, such as MESA, traditional HRV indices may fail to reflect accurately changes in cardiac vagal tone*.

We introduced fragmentation analysis to quantify short-term HR dynamics in such types of cohorts. The term “fragmented heart rate” refers to rhythms in which HR acceleration sign changes at a frequency higher than that attributable to healthy vagal tone modulation of the SAN. These anomalous rhythms include but are not limited to classic sinus alternans and its variants. Due to their anti-correlated structure, i.e., the fact that an increase in HR is likely followed by a decrease and *vice-versa*, fragmented sinus rhythms are not random/erratic. In fact, they exhibit a much higher degree of predictability/regularity (Figure 1, bottom panels) than random rhythms. The term “fragmented” was also chosen to help convey the putative pathophysiologic concept of regulatory network disintegration and/or breakdown of neuroautonomic coupling between heart rate and respiration.

An intuitive measure of fragmentation is the percentage of changes in HR acceleration sign, that is, PIP, in NN (or RR) time series. Most recently, a symbolic dynamical approach was introduced (Costa et al., 2017b) that quantifies the frequency of occurrence of different patterns of fluctuations, from least fragmented (most fluent) to most fragmented. These fragmentation indices were originally tested in studies of publicly available databases from the Rochester THEW archives. Of note, if the amplitude of the fluctuations is low (e.g., ≲80ms), fragmentation is unlikely to be detected in clinical readings of short (typically 10 s) and long (Holter) ECG recordings. However, as shown in Figure 1, visual detection of HRF is facilitated by inspection of HR time series.

The origins of HRF remain speculative. Possible pathophysiologic mechanisms include increased automaticity in or proximal to the SAN, exit block in the SAN area, modulated sinus/atrial parasystole, abrupt pacemaker shifts in the SAN (Boyett et al., 2000; Kodama et al., 2004), beat-to-beat changes due to perturbations in atrial stretch receptors (Costa et al., 2017a,b) or alterations in membrane and cellular pacemaker clocks (Lakatta et al., 2010). These electrophysiologic perturbations, in turn, may be related to underlying atrial (Sosnowski and Petelenz, 1995; Roberts-Thomson et al., 2007; Goette et al., 2017) or ventricular disease. Systemic and local factors that may also contribute to pathophysiologic dysregulation of SAN dynamics include inflammation, degeneration, fibrosis and calcification (Costa et al., 2017a). Future experimental and mathematical modeling studies will hopefully shed light on the putative links between these and other mechanisms and fragmentation. Possible genomic associations with HRF remain to be explored.

Based on the analyses of the THEW databases (Costa et al., 2017a,b), we hypothesized that increased HRF might be a biomarker of increased risk of incident CVEs and CV death. To explore these hypotheses, we analyzed HR dynamics from a subset of the participants in the MESA. This national study is one of the largest prospective investigations designed to track meticulously the course of CVD in an ethnically diverse population free of overt clinical CVD at study entry. Two types of ECG recordings with detailed follow-up data were available at the time of this analysis: traditional 10-s ECGs and the ECG channel of the PSG studies. We chose to examine the latter given the non-stationary nature of HR dynamics, [which implies that statistical time series analysis tools are most reliable when applied to “long” recordings (HRV, 1996)], and the fact that previous studies (Costa et al., 2017a,b) have shown that the discriminatory power of HRF was generally comparable during awake and sleep periods. In MESA, we confirmed and extended this initial observation. Specifically, we found that the positive association between HRF and incident CVEs (in fully adjusted analyses) could be detected for each of the sleep stages and for awake periods before sleep onset and/or after sleep termination.

A concomitant finding was the absence of associations between the most commonly used traditional short-term HRV measures and incident CVEs and CV death, in unadjusted and adjusted models. These results are not as surprising as they might appear at first glance. First, the U-shaped relationship between traditional short-term HRV measures and the participants' age (Figure 2) was indicative that such measures would be of limited utility in this cohort. Second, as previously mentioned, traditional measures of HRV, in contrast to fragmentation measures, also failed to discriminate patients with CAD from ostensibly healthy subjects in databases provided by the University of Rochester (Costa et al., 2017a,b). Third, HRF, by increasing variability not ascribable to physiologic vagal tone modulation may confound the results of traditional HRV. The nonlinear (U-shaped) relationship between HRF and short-term variability (Figure 4) supports this conjecture. In fact, the subgroups of participants with the lowest and highest amounts of variability, which, in the conventional HRV framework, would be presumed to have the highest and lowest risk of adverse events, respectively, both showed increased HRF. These findings are consistent with the reports of Stein et al. (Stein, 2002; Stein et al., 2008) and others (de Bruyne et al., 1999; Huikuri and Stein, 2012; Drawz et al., 2013).

Of note, fragmentation and traditional HRV indices differ in the following major way. By construction, fragmentation indices do not mathematically depend on mean HR and/or the amplitude of its fluctuations. These salient attributes derive from the fact that accelerations/decelerations are defined as increments/decrements in HR of any magnitude. In contrast, by definition, short-term HRV indices quantify information that is encoded in the amplitude of the fluctuations. As previously mentioned rMSSD, pNN50, and HF power were not associated with risk of incident CVEs and CV mortality. The other widely used HRV metrics, AVNN, SDNNIDX, and LF/HF were also not associated with risk of incident CVEs and CV mortality. Furthermore, none of the traditional indices improved the performance of models that included a fragmentation index.

In this study the short-term DFA index, α_1_, decreased with the participants' age. In addition, α_1_ was lower in those with adverse events (incident CVEs and CV death) than in those without. However, the associations were *not* statistically significant in any of the models.

Of potential basic and translational importance is the fact that we used both the NN and RR time series in fragmentation analyses. The NN series were employed using expert edited time series from THEW and MESA to insure that the fragmentation was likely related to beats originating in or near to SAN, therefore not distinguishable from sinus beats, at least from the single lead provided. The RR time series were used to demonstrate that fragmentation analysis, not relying on detailed beat annotation, had comparable (or even superior) discriminatory power to that employing NN time series, substantially facilitating the development of automatable analyses.

Finally, it is worth emphasizing that the Framingham and the MESA indices are composite measures incorporating information related to demographics (age, sex, race), lifestyle (smoking status), vital signs (blood pressure) and blood analytes (lipids, glucose). In contrast, each HRF index is a dynamical measure reflecting the frequency of the changes in HR acceleration sign. How can such a single metric based on a continuous ECG keep “pace” with these other multivariable risk stratification tools? The answer may relate in part to the fact that HRF indices are *dynamical* measures, not *static* probes. In contrast, blood pressure, cholesterol, glucose and other common biomarkers are single time point readouts (“snapshot”). Thus, they provide limited information on the *dynamics* of the underlying control mechanisms.

More generally, HRF metrics belong to the new class of dynamical probes (Cysarz et al., 2000; Goldberger, 2006; Costa et al., 2014; Porta et al., 2015; Makowiec et al., 2015; Hoyer et al., 2017; Zhang et al., 2017) that can report, in real-time, on salient aspects of integrative, multiscale, regulatory systems and of their breakdown with aging and disease. The use of these probes may enhance the clinical utility of traditional risk assessment tools (Tables 3, 4) and of other emerging technologies, such as genomic profiling. In furtherance of the goals of precision medicine, the dynamical property of HRF may also constitute a novel “target” for therapeutic interventions.

## 5. Conclusion

HRF, a newly defined manifestation of anomalous short-term sino-atrial variability, is associated with increased risk of cardiac adverse events and cardiac mortality in MESA. The measures, readily computable from long, continuous ECGs, added value to the canonical Framingham and MESA CV risk indices. Furthermore, fragmentation measures outperformed conventional HRV measures and the short-term DFA α_1_ index. Future studies are needed to confirm the utility of HRF measures in risk stratification, and in prediction of cardiomyopathies and atrial fibrillation.

## Author contributions

The idea for this study was developed by MC and AG. Data acquisition was the responsibility of SR. The analysis of the data was performed by MC. RD and SH directed the statistical analysis. All co-authors participated in the interpretation of the findings. MC and AG wrote the manuscript in collaboration with the other co-authors.

### Conflict of interest statement

The authors declare that the research was conducted in the absence of any commercial or financial relationships that could be construed as a potential conflict of interest.

## Abbreviations

AVNN: average value of the NN intervals
BMI: body mass index
BP: blood pressure
CAD: coronary artery disease
CV: cardiovascular
CVD: cardiovascular disease
CVE: cardiovascular event
DFA: detrended fluctuation analysis
ECG: electrocardiogram
HDL: high density lipoprotein
HF: total spectral power of all NN intervals between 0.15 and 0.4 Hz
HR: heart rate
HRF: heart rate fragmentation
HRV: heart rate variability
LF/HF: ratio of low to high frequency power
MESA: Multi-Ethnic Study of Atherosclerosis
NN: normal-to-normal sinus (interval)
PIP: percentage of inflection points (changes in heart acceleration sign)
pNN50: percentage of differences between adjacent NN intervals that are greater than 50 ms
PSG: polysomnography or polysomnogram
rMSSD: square root of the mean of the squares of differences between adjacent NN intervals
RR: R-to-R (interval)
SAN: sino-atrial node
SD: standard deviation
SDNNIDX: mean of the standard deviations of NN intervals in all 5-min segments
W_*j*_: segment, termed “word”, of 4 consecutive differences between adjacent interbeat intervals presenting *j* changes in heart rate acceleration sign.
